# The American Year Book of Medicine and Surgery

**Published:** 1903-05

**Authors:** 


### Books and Magazines.

**All books intended for review in the Texas Medical Journal must be sent to Associate Editor Dr. W. B. Russ, San Antonio, Texas.**

---

THE AMERICAN YEAR BOOK OF MEDICINE AND SURGERY.—A yearly Digest of Scientific Progress and Authoritative Opinions in all branches of Medicine and Surgery, drawn from journals, monographs, and text-books of the leading American and foreign authors and investigators. Arranged, with critical editorial comments, by eminent American specialists, under the editorial charge of George M. Gould, A. M., M. D. In two volumes—Volume I, including General Medicine, octavo, 700 pages, fully illustrated; Volume II, General Surgery, octavo, 670 pages, fully illustrated. Philadelphia, New York, London: W. B. Saunders & Co. 1903. Per volume: Cloth, $3.00, net; half Morocco, $3.75, net.

Every physician and surgeon who desires to keep fully abreast with the times, should read "Gould's Year-Book of Medicine and Surgery." Today there are so many good journals worth reading that the busy doctor has not the time to read them all, and the eminent authorities whom Dr. Gould has selected as his assistants, carefully dissect out the good and scientific matter and so present it as to render these volumes not only valuable but almost indispensable.

Volume I deals with General Medicine, and volume II General Surgery. As usual, its illustrative feature is well taken care of, there being eleven full-page inserts, besides many excellent text cuts. T. P. L.

